# Modelling the dynamics of change in the technical skills of young basketball players: The INEX study

**DOI:** 10.1371/journal.pone.0257767

**Published:** 2021-09-22

**Authors:** Eduardo Guimarães, Adam D. G. Baxter-Jones, A. Mark Williams, Fernando Tavares, Manuel A. Janeira, José Maia

**Affiliations:** 1 Centre of Research, Education, Innovation and Intervention in Sport (CIFI^2^D), Faculty of Sport, University of Porto, Porto, Portugal; 2 College of Kinesiology, University of Saskatchewan, Saskatoon, Canada; 3 Department of Health and Kinesiology, College of Health, University of Utah, Salt Lake City, Utah, United States of America; Universidade de Tras-os-Montes e Alto Douro, PORTUGAL

## Abstract

Although technical skills are a prerequisite for success in basketball, little is known about how they develop over time. In this study, we model the trajectories of technical skill development in young basketball players and investigate the effects of training experience, training volume, body composition, maturity status, physical performance, and club characteristics on skill development. A total of 264 male basketballers from five age-cohorts (11 to 15 years of age) were followed consecutively over three years using a mixed-longitudinal design. Technical skills, training experience and volume, basic anthropometrics, body composition, biological maturation and physical performance were assessed bi-annually. A multilevel hierarchical linear model was used for trajectory analysis. Non-linear trends (*p* < 0.01) were observed in speed shot shooting, control dribble, defensive movement, slalom sprint, and slalom dribble. Being more experienced and physically fitter had a significant (*p* < 0.05) positive effect on technical skill development; greater fat-free mass negatively affected skills demanding quick running and rapid changes of direction with or without the ball (*p* < 0.05). Training volume and biological age did not explain differences in technical skill development (*p* > 0.05). Moreover, belonging to different clubs had no significant influence on the technical skills trajectories of players. Our findings highlight the important role that individual differences play, over and beyond club structure, in developing skills. Findings improve our understanding on how technical skills develop during adolescence through training, growth, and biological maturation.

## Introduction

High levels of technical skill are a prerequisite for success in both adult and children’s basketball, specifically with regards to actions such as catching, passing, dribbling, shooting, and shuffling [1]. At the highest competitive level, these technical skills are crucial to performance [2]. Thus, it is important to monitor the development of technical skills in young players, especially during periods of rapid growth, to ensure appropriate responses to training and competition [3]. Although the physical, physiological, tactical, and psychological attributes of young players are very important [4], coaches and club managers know that adequate levels of technical skill are a prerequisite for success [1] and are increasingly important at higher levels in team sports [5].

Previous published reports in youth sports acknowledge that basketball training and competition demands are elevated with increasing competitive level [6, 7], and that older players outperform their younger peers [8]. Furthermore, years of formal training, as well as the number of weekly training hours, positively impact skill development [9]. More experienced players tend to perform better, especially in offensive skills involving ball handling, such as, shooting, passing, and dribbling tasks [6, 10]. Body composition is also linked to skill development. Although fat-free mass has been positively related to technical skill levels [6], te Wierike et al. [11] reported a negative association between higher values of lean body mass, in forwards and centers, and dribbling performances. It has also been reported that physically fit young players tend to be more skilled [7]. This latter finding is expected given that most basketball-specific skills involve multidirectional movements and intense motor tasks, like jumping, sprinting, and changes of direction, all of which are associated with physical fitness levels [12, 13]. It is also well known that biological maturation plays an important role in the developmental trajectories of young players, especially during adolescence when growth is most rapid [14]. However, previously researchers focusing on game-related skills have produced inconsistent findings; some reported little or no impact of biological maturation on technical skills [11], whereas others have found significant relationships, particularly for defensive skills [10].

While the importance of cross-sectional information is acknowledged, it is limited by its inability to derive developmental trajectories and inter-individual differences [15]. This issue is important given the fact that individuals show different rates of growth and maturation. It has also been reported previously that longitudinal data are more valuable when they are related to physical performance rather than technical skill development. For example, te Wierike et al. [16] used a 2-year mixed-longitudinal design to investigate the development of repeated sprint ability, and other related factors, in 14-19-year-old basketball players. The ability to perform repeated sprints developed most rapidly from 14 to 17 years of age, followed by a plateau until 19 years of age. Moreover, repeated sprint ability was positively affected by chronological age, lower body explosive strength and interval endurance capacity. Additionally, Carvalho et al. [17] examined longitudinal changes in functional performance between 12–16 years and reported a non-linear trend in countermovement jump and line drill tests, with the rate of improvement decreasing at approximately 14 years of age. The age of fourteen years typical aligns with the adolescent growth spurt. In contrast, a linear trend was found in Yo-Yo Intermittent Recovery Test—Level 1 and in an overall performance index. Changes in functional performance were positively influenced by age and years of formal training experience and negatively influenced by adiposity.

Sekine et al. [18], using a pre-post design to study muscle morphology, jump and sprint performance in 12-15-year-old basketballers, reported that after one year players advanced in maturation improved significantly more in jump and sprint performance when compared to average and late maturing players. A study of technical skill (ball control) relating to self-regulatory skills in adolescent basketball players aged 13–20 years [19] found that ball control improved with age, especially between 13 and 17 years. Ball control interacted with positional differences, with guards revealing the highest rate of ball control development over the same ages.

It is widely understood that basketball requires high levels of proficiency in a wide range of technical skills at all levels and competitive age periods. It is acknowledged that “individual basketball skills should be the starting point for every coach” [1]. Players spend many hours within their club’s environment learning and enhancing skills, implicating an important role for the club environment [20]. Based on Bronfenbrenner’s [21] ecological theory and a multilevel modelling statistical framework, we designed an integrated approach to investigate technical skill development in young basketballers in an effort to better understand the trajectories of development.

We had two aims. First, we modelled and compared technical skills and intra-individual developmental trajectories in young basketball players. Second, we examined the effects of time-varying covariates on skill trajectories. These covariates included training experience, training volume, body composition, biological age, physical performance, and club characteristics. We hypothesize that technical skills develop in a linear manner, with a constant rate of change and that increases in training experience, training volume, fat-free mass, biological maturation, and physical performance improve skill development. We also hypothesize that the characteristics of each club will be significantly associated with skill development.

## Materials and methods

### Sample and design

Players were selected from the *In Search of Excellence—a Mixed-longitudinal Study in Young Athletes* (INEX) study (2017–2019). This three-year mixed-longitudinal design employed five age-cohorts (11, 12, 13, 14, and 15 years) that over the course of three years had a two-year overlap between cohorts. Using such a design it was possible to create seven-year developmental trajectories from three years of data collection. The main aim of the INEX study was to investigate the interactions among individual characteristics and environmental factors affecting player growth, physical performance, specific-skills performance, game proficiency, and psychological development. The INEX study design is described in detail elsewhere [22].

In the present study, data on 293 adolescent male basketball players (from all five age-cohorts) were collected bi-annually over three consecutive years. Basketball players were recruited from a population of 1256 adolescent male players and were members of 20 out of the 25 clubs in the Porto Basketball Association. Participants were randomly selected to participate by their coaches and/or club team managers. The proportion of dropouts across the study was 6%. To be included in the present analysis, complete data were required from a minimum of two, and maximum of six, time points. In total, 264 players fulfilled this condition. In cohort 1 (*n* = 38), players were followed consecutively from 11 to 13.5 years, in cohort 2 (*n* = 53) from 12 to 14.5 years, in cohort 3 (*n* = 48) from 13 to 15.5 years, in cohort 4 (*n* = 60) from 14 to 16.5 years, and in cohort 5 (*n* = 65) from 15 to 17.5 years. Players over 18 years of age were excluded. Baseline measurements were initiated in June 2017 and repeated bi-annually until December 2019. All assessments occurred during the same time-periods (June and December) within a time window of 15–20 days. In total, 1261 measurements were obtained from 264 individuals (Table 1). Participants were divided into groups of 4–5 players and sequentially assessed in the 20 m sprint, slalom sprint, slalom dribble, speed shot shooting, countermovement jump, 3 kg seated medicine ball throw, defensive movement, passing, T-test, and control dribble tests. We obtained written informed consent from parents or legal guardians as well as individual assent from each basketball player. The Ethics Committee of the lead Faculty (CEFADE 13.2017) approved the study, and the Porto Basketball Association gave formal permission for data collection.

**Table 1:** Number of measurements per time-point for each age category.

Age (years)	Time-points						Total
	June 2017	December 2017	June 2018	December 2018	June 2019	December 2019	
11.0 (10.50–10.99)	11						11
11.5 (11.00–11.49)	27	7					34
12.0 (11.50–11.99)	22	22	9				53
12.5 (12.00–12.49)	31	17	22	11			81
13.0 (12.50–12.99)	22	24	18	25	9		98
13.5 (13.00–13.49)	23	21	29	19	23	9	124
14.0 (13.50–13.99)	25	19	21	29	18	22	134
14.5 (14.00–14.49)	23	24	22	20	28	16	133
15.0 (14.50–14.99)	11	24	20	22	18	28	123
15.5 (15.00–15.49)	23	17	22	28	21	17	128
16.0 (15.50–15.99)		25	25	25	23	20	118
16.5 (16.00–16.49)			32	23	24	23	102
17.0 (16.50–16.99)				30	22	23	75
17.5 (17.00–17.49)					25	22	47
Total measurements	218	200	220	232	211	180	**1261**

### Technical skills

Technical skills were assessed using four basketball-specific tests developed by the American Alliance for Health, Physical Education, Recreation and Dance (AAHPERD) [23], and two slalom tests developed by Lemmink et al. [24]:

1. *speed shot shooting (points)*—players shot the ball from five positions (under angles of 0°, 45°, and 90° to the basket backboard) from a distance of 4.57 m, collected their own rebound, dribbled to another designated position, and repeated this sequence as quickly as possible over 60 s. A maximum of four non-consecutive lay-ups were allowed during each trial. Successful shots counted as two points while each unsuccessful one that hit the rim from above counted as one point. Each player performed three trials including one practice trial. The sum of the second and third trials was used as the test result.
2. *passing (points)*—players performed chest passes against a wall marked with six specific targets of 60×60 cm with alternating bases 150 cm and 90 cm from the floor, and retrieved the ball while moving laterally behind a restraining line 2.45 m from the wall over 30 s. Each pass hitting the target or the boundary counted as two points, while those hitting the intervening spaces on the wall counted as one point. Each player performed three trials including one practice trial. The sum of the second and third trials was used as the test result.
3. *control dribble (s)*—players dribbled the ball while running as quickly as possible over an obstacle course, defined by six cones placed within the basketball restricted area measuring 5.8 m×3.6 m. The time taken to complete the test was recorded. Each player performed three trials including one practice trial. The sum of the second and third trials was used as the test result.
4. *defensive movement (s)*—while keeping the basic defensive position, players performed as quickly as possible lateral slides without crossing their feet in a sequence of seven changes of direction, defined by six cones placed in the lines limiting the basketball restricted area. The time taken to complete the test was recorded. Each player performed three trials including one practice trial. The sum of the second and third trials was used as the test result.
5. *slalom sprint (s)*—players had to run and change direction as quickly as possible in a zig-zag pattern defined by twelve cones over a 29.07 m course. The time taken to complete the test was recorded. Each player performed two trials and the best one was used as the test result.
6. *slalom dribble (s)*—players had to dribble and control the ball while running and changing direction as quickly as possible in a zig-zag pattern defined by twelve cones over a 29.07 m course. The time taken to complete the test was recorded. Each player performed two trials and the best one was used as the test result.

### Training information

The players’ training experience, expressed as years of formal basketball training, was obtained from self-report questionnaires, confirmed by questioning coaches and/or club team managers, and validated against registration histories available from the official website of the Portuguese Basketball Federation (FPB; http://www.fpb.pt). To be granted permission to compete in Portugal, players are required to register in the FPB. A player registered for one competitive season would have one year of training experience, a player registered for two competitive seasons, two years of training, and so on. Training volume data, expressed as training hours per week, were obtained from self-report questionnaires and confirmed by oral questioning of coaches and/or club team managers. During the study, all under-12, under-14, and under-16 players regularly trained 4.5 h·week^–1^, whereas under-18 players trained regularly 6.0 h·week^–1^. In the analysis, 4.5 h·week^–1^ was the reference category.

### Anthropometry and body composition

Height (cm) and sitting height (cm) were measured using a Harpenden stadiometer (Holtain Ltd., Crymych, UK) without shoes and with the participant’s head positioned in the Frankfurt plane. The precision was 0.1 cm. Body mass (kg) was measured using a bio-impedance scale (Tanita®BC-418MA, Tanita Corp., Tokyo, Japan) with a precision of 100 g, and fat-free mass (kg) was derived according to the manufacturer’s formula for athletes. All measurements were taken by experienced anthropometrists following the International Working Group on Kinanthropometry protocols [25].

### Biological maturation

Biological maturation was assessed by predicting years from attainment of peak height velocity (PHV), a biological age in years, using a maturity prediction equation from anthropometric data [26]. The equation uses sex-specific measures of chronological age, height, sitting height and body mass to predict years from or after the occurrence of PHV, termed as “maturity offset (years from PHV)”. A positive (+) maturity offset represents the predicted number of years the participant is beyond their age of PHV, whereas a negative (–) value represents the predicted number of years before the attainment of their PHV.

### Physical performance

Physical performance was assessed using four standardized tests:

1. *countermovement jump (cm)*—players performed a vertical jump, as advocated by Bosco et al. [27], on a AMTI OR6-WP force platform (Advanced Mechanical Technology Inc., Watertown, MA, USA) operating at 2000 Hz. Jumping height (cm) was estimated using the flight time method as described by Linthorne [28]. Players quickly squatted down until the knees were bent at 90° and immediately jumped vertically as high as possible, landing on both feet at the same time. Players were instructed to start standing as still as possible on the force platform with their weight evenly distributed over both feet, and to keep their hands on the hips throughout the test. Each player performed three trials and the best one was used as the test result.
2. *3 kg seated medicine ball throw (m)*—players threw the ball straight forward as far as possible while seated on the floor with their legs fully stretched and their backs against a wall. The distance from the wall to where the ball landed was recorded and the mean of three trials was used as the test result [29].
3. *20 m sprint (s)*—players stood on the 0 m mark and, when ready, ran in a straight line at full speed. Time was recorded using the photoelectric cells system Speed Trap II (Brower Timing Systems LLC., Draper, UT, USA). Each player performed two trials and the best one was used as the test result [30].
4. *T-test (s)*—players ran and changed directions rapidly in a T-shape pattern while maintaining balance and without loss of speed. Time was obtained using the photoelectric cells system Speed Trap II (Brower Timing Systems LLC., Draper, UT, USA). Each player performed two trials and the best one was used as the test result [31].

An overall measure of physical performance was used after transforming individual test results into z-scores and computing an unweighted sum of all z-scores. Care was taken to reverse signs in 20 m sprint and T-test since in both tests less time represents better performance.

### Club information

A questionnaire was used to collect detailed club information across four areas: (1) club characteristics (number of sports, number of athletes, number of basketball players, number of competitive age-categories, number of national and regional titles, and number of years since the foundation of the club’s basketball section); (2) club infrastructure (own facilities, complementary equipment, and basketball practices location); (3) human resources (number of coaches, coaches’ certification level, and staff); and (4) club communication (social media, and radio station or tv/online channel). All club presidents or directors answered the questionnaire during visits to the club facilities under the supervision of a trained member of the research team. The characteristics of the twenty clubs are presented in S1 Table in S1 File.

### Data quality control

A five-step procedure was used to ensure data quality control: (1) anthropometric measurements were performed by trained personnel from the Kinanthropometry Lab of the lead Faculty; (2) an in-field reliability approach was used such that a random sample of three-to-five participants were re-measured every day; (3) reliability estimates were computed using the technical error of measurement (TEM) as well as an ANOVA-based intraclass correlations (R). The TEM was 0.2 cm for height, 0.1 cm for sitting height, 0.1 kg for body mass, and 0.3 kg for fat-free mass. Furthermore, for technical skills tests, R-values ranged from 0.91 (speed shot shooting) to 0.98 (defensive movement), whereas R-values for physical performance tests ranged from 0.93 (countermovement jump) to 0.99 (3 kg seated medicine ball throw); (4) data cleaning was undertaken to control for punching errors in data entry as well as the putative presence of outliers; finally, (5) normality checks in the distributions of all variables were undertaken.

### Statistical analysis

All normality checks and descriptive statistics (Mean ± SD; Counts and percentages) were performed using IBM SPSS 26.0 (IBM Corp., Armonk, NY, USA). Given the hierarchical structure of the data, that is, repeated observations (level-1) nested within players (level-2) which are themselves nested within clubs (level-3), multilevel linear regression models were used [32]. The time metric was centered around 11 years of age to ensure the intercept was centered within the data set. Thus, the metric of time of 0, 0.5, 1, 1.5, 2, 2.5, 3, 3.5, 4, 4.5, 5, 5.5, 6, 6.5, corresponds to 11, 11.5, 12, 12.5, 13, 13.5, 14, 14.5, 15, 15.5, 16, 16.5, 17, 17.5 years of age respectively. Models were developed using a two-step approach. To test the first hypothesis, polynomials of age were added, namely, age and age^2^ (S2-S7 Tables in S1 File). Then, in each technical skill the following set of time dependent predictors were added: cohort effects because of the mixed-longitudinal design; training experience; training volume; fat-free mass; maturity offset; and overall physical performance. To test the second hypothesis, level-3 variance (club level) was freely estimated. Parameters were simultaneously estimated using a full maximum likelihood procedure implemented in SuperMix 2.0 software (Scientific Software International, Inc., Lincolnwood, IL, USA) [33]. This procedure is robust, and all parameter estimates are precise and consistent. Additionally, residuals were inspected and the significance level was set at 5%.

## Results

The descriptive statistics for technical skills, training information, body composition, biological maturation, as well as physical performance are shown in Table 2. In general, players became more skillful with increasing age. The mean outcomes in speed shot shooting and passing tests increased with age, whereas in control dribble, defensive movement, slalom sprint, and slalom dribble timed performance systematically improved with age. In addition, mean training experience, fat-free mass, maturity offset (changing from negative to positive values), and overall physical performance increased with age, whereas mean training volume showed a distinct pattern, with the number of training hours per week being the same until 16.0 years of age and then increasing.

**Table 2:** Descriptive statistics (Mean ± SD) for all age groups.

**Variables**	**11.0 years**	**11.5 years**	**12.0 years**	**12.5 years**	**13.0 years**	**13.5 years**	**14.0 years**
	**(*n =* 11)**	**(*n =* 34)**	**(*n =* 53)**	**(*n =* 81)**	**(*n =* 98)**	**(*n =* 124)**	**(*n =* 134)**
**Technical skills**							
Speed shot shooting (points)	21.18 ± 7.72	25.44 ± 5.77	28.09 ± 5.83	29.05 ± 6.01	30.71 ± 5.28	32.02 ± 5.64	33.74 ± 5.10
Passing (points)	70.91 ± 12.94	73.79 ± 11.62	78.70 ± 10.30	78.14 ± 9.81	85.25 ± 10.51	88.73 ± 11.30	93.11 ± 11.77
Control dribble (s)	22.21 ± 1.45	21.14 ± 1.84	20.36 ± 1.53	20.64 ± 1.62	19.81 ± 1.36	19.30 ± 1.30	18.75 ± 1.25
Defensive movement (s)	26.92 ± 2.06	26.64 ± 1.92	25.56 ± 2.11	25.00 ± 2.04	23.67 ± 1.84	22.85 ± 1.65	22.42 ± 1.64
Slalom sprint (s)	18.56 ± 0.98	18.12 ± 1.08	17.85 ± 0.90	17.66 ± 1.17	17.03 ± 0.98	16.80 ± 1.00	16.45 ± 0.90
Slalom dribble (s)	20.65 ± 1.28	19.52 ± 1.41	19.17 ± 1.11	18.91 ± 1.43	18.26 ± 1.19	17.80 ± 1.19	17.44 ± 1.02
**Training information**							
Training experience (years)	2.91 ± 1.45	3.22 ± 1.33	3.74 ± 1.47	3.74 ± 1.52	4.40 ± 1.56	4.66 ± 1.67	4.94 ± 1.69
Training volume (h·week^–1^)	4.50 ± 0.00	4.50 ± 0.00	4.50 ± 0.00	4.50 ± 0.00	4.50 ± 0.00	4.50 ± 0.00	4.50 ± 0.00
**Body composition**							
Fat-free mass (kg)	29.65 ± 2.85	30.92 ± 3.98	33.81 ± 4.67	37.10 ± 5.79	40.37 ± 6.74	43.27 ± 7.13	46.19 ± 7.04
**Biological maturation**							
Maturity offset (years)	–2.61 ± 0.33	–2.25 ± 0.41	–1.84 ± 0.42	–1.29 ± 0.55	–0.82 ± 0.60	–0.36 ± 0.68	0.15 ± 0.64
**Physical performance**							
Overall physical performance (z-score)	–6.06 ± 1.72	–5.65 ± 1.88	–4.98 ± 2.26	–3.85 ± 2.26	–2.75 ± 2.33	–1.70 ± 2.36	–0.75 ± 2.32
	**14.5 years**	**15.0 years**	**15.5 years**	**16.0 years**	**16.5 years**	**17.0 years**	**17.5 years**
	**(*n =* 133)**	**(*n =* 123)**	**(*n =* 128)**	**(*n =* 118)**	**(*n =* 102)**	**(*n =* 75)**	**(*n =* 47)**
**Technical skills**							
Speed shot shooting (points)	34.66 ± 5.28	36.35 ± 4.74	36.49 ± 4.63	37.38 ± 4.48	38.67 ± 5.29	39.30 ± 5.83	39.87 ± 5.10
Passing (points)	95.88 ± 12.59	99.01 ± 12.03	103.83 ± 12.95	105.82 ± 12.76	108.73 ± 13.68	111.27 ± 14.45	114.84 ± 13.12
Control dribble (s)	18.38 ± 1.08	18.02 ± 1.14	18.03 ± 1.01	17.82 ± 1.04	17.53 ± 1.08	17.35 ± 1.06	17.00 ± 1.01
Defensive movement (s)	21.93 ± 1.65	21.31 ± 1.50	21.20 ± 1.36	20.73 ± 1.31	20.46 ± 1.21	20.11 ± 1.44	20.34 ± 1.48
Slalom sprint (s)	16.27 ± 0.86	16.01 ± 0.96	15.98 ± 0.87	15.79 ± 0.83	15.46 ± 0.79	15.37 ± 0.80	15.52 ± 0.98
Slalom dribble (s)	17.09 ± 0.95	16.95 ± 1.11	16.79 ± 0.91	16.63 ± 0.93	16.32 ± 1.06	16.32 ± 0.93	16.23 ± 1.12
**Training information**							
Training experience (years)	5.32 ± 1.92	5.61 ± 2.03	6.15 ± 2.03	6.58 ± 2.16	7.04 ± 2.18	7.75 ± 2.21	8.13 ± 2.16
Training volume (h·week^–1^)	4.50 ± 0.00	4.50 ± 0.00	4.50 ± 0.00	4.50 ± 0.00	5.15 ± 0.75	5.98 ± 0.17	6.00 ± 0.00
**Body composition**							
Fat-free mass (kg)	49.14 ± 7.44	51.60 ± 7.47	53.96 ± 7.67	54.76 ± 6.95	57.67 ± 6.73	59.31 ± 6.68	65.63 ± 6.72
**Biological maturation**							
Maturity offset (years)	0.63 ± 0.63	1.11 ± 0.68	1.50 ± 0.73	1.92 ± 0.64	2.42 ± 0.67	2.71 ± 0.54	3.12 ± 0.59
**Physical performance**							
Overall physical performance (z-score)	0.29 ± 2.10	0.90 ± 2.19	1.82 ± 2.03	2.35 ± 2.14	3.08 ± 2.15	3.68 ± 2.15	3.82 ± 1.85

**Fig 1:**
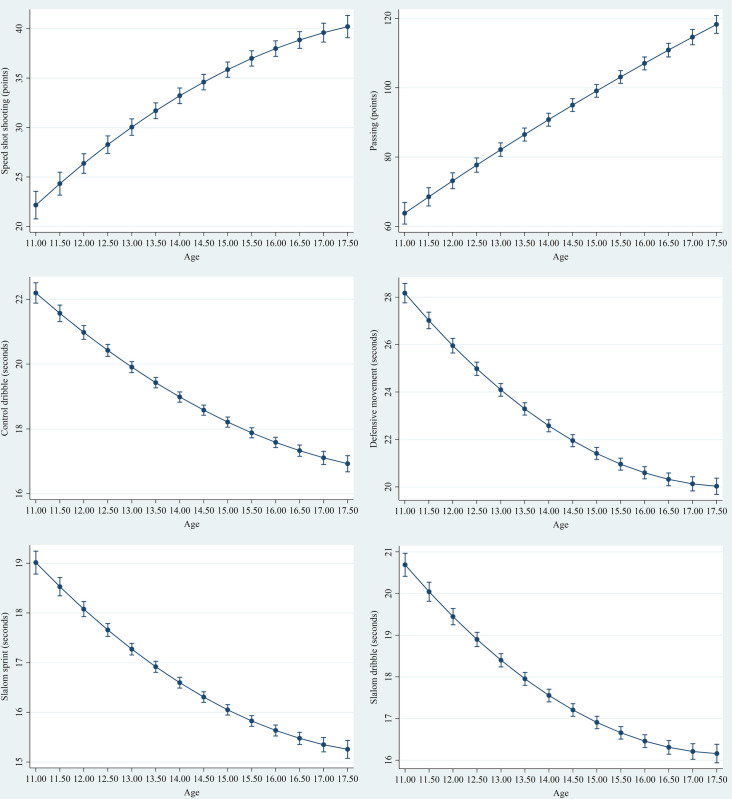
Technical skills trend lines with 95% confidence intervals.

The results of the multilevel models are shown in Table 3. The regression coefficients showed that, on average, an 11-year-old basketball player (see intercept in fixed effects in Table 3) has a speed shooting score of 26.02 ± 1.23 points, a passing score of 80.34 ± 2.44 points, a control dribble score of 20.69 ± 0.26 s, a defensive movement of 25.72 ± 0.31 s, a slalom sprint of 18.12 ± 0.19 s, and a slalom dribble of 19.62 ± 0.23 s (*p* < 0.001). A significant non-linear positive trend was observed in speed shot shooting (β = –0.22 ± 0.07, *p* < 0.01), and a significant non-linear negative trend was observed in control dribble (β = 0.04 ± 0.02, *p* < 0.01), defensive movement (β = 0.15 ± 0.02, *p* < 0.001), slalom sprint (β = 0.06 ± 0.01, *p* < 0.001), and slalom dribble (β = 0.09 ± 0.01, *p* < 0.001), all improved with increasing age (Fig 1). Cohort effects were all significant in all skill tests (*p* < 0.01). Players with more training experience were, on average, more skillful with increasing age [speed shot shooting (β = 0.58 ± 0.11, *p* < 0.001), passing (β = 1.81 ± 0.27, *p* < 0.001), control dibble (β = –0.08 ± 0.02, *p* < 0.001), defensive movement (β = –0.06 ± 0.03, *p* < 0.05), and slalom dribble (β = –0.08 ± 0.02, *p* < 0.001)]. In contrast, training volume did not explain differences in developmental trajectory on any technical skill (*p* > 0.05). Furthermore, basketball players with greater fat-free mass needed more time to complete the control dribble (β = 0.02 ± 0.01, *p* < 0.05), slalom sprint (β = 0.02 ± 0.01, *p* < 0.01), and slalom dribble (β = 0.02 ± 0.01, *p* < 0.05) tests. Biological age (maturity offset) was not significantly independently associated with the developmental trajectories of any technical skills (*p* > 0.05), but physically fitter players were more skillful [speed shot shooting (β = 0.35 ± 0.08, *p* < 0.001), passing (β = 1.57 ± 0.18, *p* < 0.001), control dibble (β = –0.26 ± 0.02, *p* < 0.001), defensive movement (β = –0.36 ± 0.02, *p* < 0.001), slalom sprint test (β = –0.19 ± 0.01, *p* < 0.001), and slalom dribble (β = –0.21 ± 0.02, *p* < 0.001)].

**Table 3:** Multilevel regression models for technical skills development: Parameter estimates (standard-errors) for fixed and random effects.

	Speed shot shooting (points)	Passing (points)	Control dribble (s)	Defensive movement (s)	Slalom sprint (s)	Slalom dribble (s)
**Fixed effects, β**						
Intercept (11 years)	26.02 (1.23)***	80.34 (2.44)***	20.69 (0.26)***	25.72 (0.31)***	18.12 (0.19)***	19.62 (0.23)***
Age (velocity)	3.30 (0.55)***	4.41 (0.77)***	–0.76 (0.12)***	–1.58 (0.14)***	–0.71 (0.09)***	–1.00 (0.10)***
Age^2^ (acceleration)	–0.22 (0.07)**	----------	0.04 (0.02)**	0.15 (0.02)***	0.06 (0.01)***	0.09 (0.01)***
CE_c2-c1	–1.54 (0.58)**	–5.72 (1.20)***	0.40 (0.13)**	0.71 (0.16)***	0.46 (0.09)***	0.37 (0.11)***
CE_c3-c2	–2.82 (0.67)***	–7.43 (1.47)***	0.73 (0.14)***	0.95 (0.17)***	0.68 (0.11)***	0.57 (0.13)***
CE_c4-c3	–2.65 (0.66)***	–9.47 (1.50)***	0.99 (0.14)***	1.06 (0.17)***	0.72 (0.10)***	0.69 (0.12)***
CE_c5-c4	–2.27 (0.51)***	–7.02 (1.18)***	0.64 (0.10)***	0.60 (0.12)***	0.47 (0.08)***	0.44 (0.09)***
Training experience (years)	0.58 (0.11)***	1.81 (0.27)***	–0.08 (0.02)***	–0.06 (0.03)*	–0.03 (0.02)^ns^	–0.08 (0.02)***
Training volume (h·week^–1^)	0.02 (0.60)^ns^	–1.60 (1.00)^ns^	–0.06 (0.13)^ns^	–0.15 (0.15)^ns^	–0.13 (0.10)^ns^	–0.07 (0.11)^ns^
Fat-free mass (kg)	0.06 (0.04)^ns^	0.14 (0.10)^ns^	0.02 (0.01)*	0.01 (0.01)^ns^	0.02 (0.01)**	0.02 (0.01)*
Maturity offset (years)	–0.11 (0.47)^ns^	0.04 (1.04)^ns^	–0.01 (0.10)^ns^	–0.14 (0.12)^ns^	–0.09 (0.08)^ns^	0.02 (0.09)^ns^
Overall physical performance (z-score)	0.35 (0.08)***	1.57 (0.18)***	–0.26 (0.02)***	–0.36 (0.02)***	–0.19 (0.01)***	–0.21 (0.02)***
**Random effects, σ** ^**2**^						
*Club*						
Intercept	0.63 (0.48)^ns^	3.48 (2.61)^ns^	0.03 (0.02)^ns^	0.06 (0.04)^ns^	0.01 (0.01)^ns^	0.02 (0.02)^ns^
*Player*						
Intercept	22.44 (5.10)***	53.21 (17.70)**	1.17 (0.25)***	1.87 (0.38)***	0.51 (0.13)***	0.97 (0.20)***
Age	1.04 (0.33)**	2.64 (1.37)^ns^	0.04 (0.01)**	0.07 (0.02)**	0.02 (0.01)**	0.04 (0.01)***
Covariance (intercept/age, σ_ia_)	–4.08 (1.28)**	–6.25 (4.81)^ns^	–0.21 (0.06)**	–0.32 (0.09)***	–0.09 (0.03)**	–0.18 (0.05)***
*Residual*						
Intercept	14.01 (0.69)***	61.78 (3.04)***	0.69 (0.03)***	0.91 (0.04)***	0.41 (0.02)***	0.51 (0.03)***
**Model summary**						
Deviance	6959.11	8764.05	3283.12	3667.86	2650.59	2967.16
Number of estimated parameters	17	16	17	17	17	17

CE, cohort effects; CE_c2-c1, overlapping effect of cohort 2 on cohort 1; ns, non-significant; age is centered at 11 years.

* *p* < 0.05.

** *p* < 0.01.

*** *p* < 0.001.

The variance component results of each test (see random effects in Table 3) showed that club environments (level-3) did not explain any of the variance in technical skill development (*p* > 0.05)—the estimate being less than twice the standard error. At level-2, players showed significant (*p* < 0.01) inter-individual differences at 11 years of age in both intercepts and slope of the line in all six tests. With the exception of the passing test, technical skill development trajectories showed significant inter-individual slope differences [speed shot shooting (σ^2^ = 1.04 ± 0.33, *p* < 0.01), control dribble (σ^2^ = 0.04 ± 0.01, *p* < 0.01), defensive movement (σ^2^ = 0.07 ± 0.02, *p* < 0.01), slalom sprint test (σ^2^ = 0.02 ± 0.01, *p* < 0.01), and slalom dribble (σ^2^ = 0.04 ± 0.01, *p* < 0.001)]. The negative covariances in the models indicated that the higher the intercept the lower the slope. Finally, apart from the passing test, more skillful players at 11 years of age tended to show greater performance in their technical developmental trajectories (*p* < 0.01).

## Discussion

This study investigated the trajectories of technical skill development in young basketball players and their relationships with time-varying individual characteristic predictors and club characteristics. It was found that technical skill improved with increasing age within individuals (level-1) and that between individuals there were significantly different intercepts and trajectories of skill development (level-2) while no significant club effect was found (level-3). Age squared, a fixed non-linear predictor, was significant and indicated that the trajectories were non-linear and so our first hypothesis, that skills show a linear increase with age, was rejected. Furthermore, we found that skills increased independently of age, training experience and physical performance. Finally, it was found that having greater fat-free mass negatively affected skills demanding quick running and rapid changes of direction both with and without the ball.

Given the mixed-longitudinal design, cohort effects were modelled and tested as advocated by Prahl-Andersen and Kowalski [34] in all skill tests. Since within each age-cohort (notwithstanding the 2-year overlap) player’s lives, educational and training histories may have impacted technical skill development, adjusting for this significant methodological constraint becomes necessary. To the best of our knowledge, this is the first time that a three-level hierarchical approach has been used to investigate the development of youth athletes; adjusting time dependent independent predictors over repeated observations within (level-1) and between (level-2) players and between clubs (level-3). Since serial data related to the unfolding of technical skills in youth basketballers are scarce, comparisons are somewhat limited to the study of te Wierike et al. [19]. This study is unique in using hierarchical modeling to gain insight into the development of a single basketball-specific technical skill—ball control.

Apart from the passing test, technical skill development followed a non-linear trend, which confirms previous longitudinal data from te Wierike et al. [19] who reported a non-linear improvement in ball control in young male basketballers between ages 13 and 17, followed by a decline in the rate of improvement until age 20. This trend is in part consistent with previous cross-sectional data from Matulaitis et al. [8]. The latter authors suggested that three periods exist for technical skill development: 7–10, 12–13 and 14–15 years of age.

Similarly, non-linear trajectories have been found in young male soccer players in dribbling, ball control and shooting [35], in dribbling speed [36], and in passing execution time and passing skill performance time [37]. In addition, non-linear trajectories have been reported in a composite skill score aggregating ball control, dribbling speed, shooting accuracy and passing [38], and in the slalom sprint and slalom dribble tests in soccer players [39]. These findings can be explained in part by, at least, five factors: (1) the “adolescent awkwardness”, a phenomenon of disruption of motor coordination causing an apparent plateau in performance in late adolescence [40]; (2) skill-related physical performance peak spurts occurring coincidently with peak height velocity or within 6 months of its attainment, that is, around 14 years of age in boys [41, 42]; (3) changes in body size and composition during puberty affecting technical development; (4) greater investment by basketball coaches in tactical strategies from under-16/under-18 age-categories onwards, accompanied by a reduction of training time exclusively dedicated to enhancing technical skills [1]; and (5) limited progression from a certain high-level of proficiency due to time and space restrictions of the testing protocols.

Training experience was positively related to changes over time across all technical skills, other than the slalom sprint test. During adolescence, more experienced basketball players scored more points in shooting and passing tests and needed less time to complete the courses in control dribble, defensive movement, and slalom dribble tests. Although comparisons are limited, such results corroborate our previous cross-sectional data from Portuguese adolescent basketball players [6, 10]. On the other hand, training volume was not significantly associated with the developmental trajectories of any technical skills. Yet, it should be noted that training volume has little variation across the study years (players always trained 4.5 h·week^–1^ from 11.0 to 16.0 years). In fact, the nature of this variable may be the reason why this finding is in contrast with previous longitudinal studies, namely from youth soccer, reporting positive associations between training hours per week or per year and dribbling speed [36], skill composite [38], and slalom dribble test [39]. Also, our findings do not align with previous cross-sectional investigations with training data collected retrospectively, which showed a positive effect of average hours per year of engagement in sport-specific activities on technical performance of alpine skiers [43] and perceptual-cognitive skills of soccer players [44]. It is possible that the uniqueness of each sample, sport specificities, as well as differences in test protocols may be responsible for these discrepancies. Notwithstanding, it would be expected that both markers of training were significantly linked with technical skill trajectories over time since more time (i.e., number of seasons or hours per week) engaged in training and competition usually represents more opportunities to players improve both offensive and defensive basketball fundamentals. Yet, as Valente-dos-Santos et al. [36] contended, “the amount of training expressed in hours is probably not a sufficiently sensitive indicator”. In fact, during practices, players are involved in distinct activities, each one with different intensity and purpose. Furthermore, the non-variation of the mean number of hours per week until the under-16 age-category can be a possible explanation for the non-significant association of training volume with technical skill development. In light of such shortcomings, we recommend that in future researchers collect data from actual training regimes.

Higher values of fat-free mass did not significantly favor shooting and passing performances over time but seemed to impact negatively on dribbling and sprinting tasks requiring rapid displacement of center of mass as well as quick sprints and changes of directions while handling the ball. Although statistically significant, the effect size expressed by the regression coefficients show an apparent “irrelevant” influence on the developmental trajectories of control dribble, slalom sprint, and slalom dribble (β = 0.02, meaning that for each increment of 1 kg of fat-free mass, players take more 0.02 s to complete the tests). The absence of similar published reports does not allow comparisons with other longitudinal data from youth basketball. Nevertheless, our findings are in agreement with previous studies in youth soccer, which found non-significant associations between fat-free mass and longitudinal trajectories in dribbling speed [36] and slalom dribble [39] tests. It is acknowledged that players with higher fat-free mass are expected to have more muscle mass and, consequently, greater explosive strength [16]. However, this is apparently not linked with technical skills performance. In any case, future researcher should give additional attention to the effects of fat-free mass on technical skill in order to better understand its role in the development of young basketballers.

There is consistent evidence of the confounding effects of biological maturation on changes in anthropometrics, body composition and physical performance development in young basketball players [6, 7] affecting team-selection and short-term participation [14]. However, our results showed that biological age (maturity offset) did not independently explain differences in developmental trajectory trends in any technical skill. These findings are in line with results from a previous longitudinal study in soccer showing no significant influence of skeletal maturity status on composite skill score [38]. Also, they confirm previous cross-sectional data reporting minor or no contribution of biological maturation to technical skills in youth basketballers [10, 11]. Given the highly specific nature of the skill testing protocols used in the present study, it is understandable that maturation per se loses importance to other factors more related to the training and competition process. Although it is expected that maturation indirectly affects basketball-specific skills via body size and physical capacities, we contend that training-related predictors such as years of previous formal training, physical performance or perceptual-cognitive skills are apparently more important to technical skill development.

Physical performance data showed that fitter basketball players tend to be more skilled across time in all six technical skill tests. Valente-dos-Santos et al. [38] reported similar findings in young soccer players followed longitudinally. Using repeated-sprint ability and aerobic endurance as functional markers, the authors reported that both capacities impacted positively on a composite of soccer-specific skills. Also, our results confirm previous cross-sectional data with young basketballers showing positive associations between functional/physiological capacities and technical skills [7, 12]. A possible explanation is that skill performance requires high levels of speed, strength, power, endurance, and agility. It is apparently simple to understand that these physical attributes fit together to make players perform the basketball-specific skills as efficiently as possible during the entire game. On the other hand, it is well-known that fatigue affects technical proficiency [45]. Thus, well prepared players in physical terms are expected to maintain good skill levels in actions like shooting, passing, rapid changing of directions while controlling the ball, as well as moving defensively.

As shown in the variance components results, no significant effects were found in technical skill development across clubs. In the present cohort, clubs did not significantly influence basketball-specific skill development over time. In other words, this novel result reveals that belonging to different clubs within the Porto Basketball Association does not impact on skill development trajectories. This finding might be in part related to the limited number of clubs considered. Furthermore, it is possible that the substantial differences observed among these clubs, namely in terms of characteristics, infrastructures, and human resources, may be surpassed by similar training regimes implemented by each coach, as well as internal policies that exist across the twenty clubs. For example, all clubs include a team manager for youth teams who was responsible not only to hire coaches with greater knowledge and competence, but also to design and implement appropriate long-term developmental programs aiming to improve, among other things, technical proficiency. Nevertheless, we recommend that in future, researchers should examine actual training programs of individual groups in sports clubs and their impact on technical skill development. In turn, since significant inter-individual differences were identified, technical skill development followed distinct paths, that is, their trajectories were not parallel. Therefore, we contend that receiving the same opportunities by the clubs does not signify that all players present the same response to training and competition across time.

This study is not without limitations. First, caution must be taken when generalizing our results as our sample is from the city of Porto, northern Portugal. Although it is expected that young basketball players from the Porto Basketball Association are relatively similar to those from other regions, we acknowledge that our sample is not widely representative. Second, we recognize that obtaining training experience information only based on years of previous formal basketball training as well as from training volume limits our specific knowledge of player career paths. Although players accumulate many hours in basketball-specific practice and competition, we recognize that time spent in play activities (e.g., game of basketball in an outdoor court with friends) was not considered in this study, and these appear to be linked to excellence in the game [44]. Yet, we believe that by consulting official records we were able to get more reliable information than we would have by using only written or oral questioning which exclusively depends on retrospective recall. Third, there will always be some shortcomings in measuring technical skills using so-called traditional skills tests. Although measuring performance during regular or small-sided games has started to be used more often, these approaches present several problematic issues: (1) the lack of consensus on how best to measure overall performance; (2) the non-availability of a standardized/validated tool; and (3) the highly time-consuming analysis of individual player performance [46]. Fourth, the reduced number of clubs considered in this study may have been responsible for the non-significant variance component. However, twenty is a number generally accepted to be included in a three-level modelling and, in our case, represented 80% of the clubs from distinct regions of the Porto city.

## Conclusions

In conclusion, our longitudinal data analysis improves current understanding of skill development in youth basketball players. In this cohort, apart from the passing test, all other technical skills unfolded in a non-linear fashion, leveling-off around late adolescence/emerging adulthood. More experienced and physically fitter players tended to be more skillful, however training volume and biological maturation had no significant links with developmental trajectories. Across age-cohorts, and over time, increases in fat-free mass tended to have a negative effect on skill development, namely in control dribble, slalom sprint, and slalom dribble tests. Finally, club environment had no apparent link with differences in technical skill progression. Since technical skills are a fundamental prerequisite to success in basketball and, as we showed, their rate of development is not constant, coaches need to be aware of this issue when planning their training schedules across competitive categories. We recommend basketball coaches to use normative assessments of technical skills to better track the developmental trajectories of their players over time. Furthermore, we suggest that coaches pay attention to physical fitness levels because they affect technical skill development. Since changes in fat-free mass are predictors of skill development, daily training routines should consider this fact together with strength training programs. We recommend that in future researchers include more detailed information about previous and current sport engagement. We also urge investigators to take account of data concerning gross motor coordination developmental levels. Additionally, information from parents and coaches as members of the sporting environment should be considered, in order to provide a more encompassing understanding on technical skill development in youth basketball players.

## Supporting information

S1 File S1-S7 Tables. (PDF)
