# Supplementary material for: Modelling the dynamics of change in the technical skills of young basketball players: The INEX study
Source: PLoS One. 2021 Sep 22;16(9):e0257767. doi: 10.1371/journal.pone.0257767 (PMC8457466; doi:10.1371/journal.pone.0257767)
Supplement: S1 File — (PDF) [file pone.0257767.s001.pdf]

S1 Table. Descriptive statistics for the clubs.

**Clubs (*n* = 20)**	**Mean ± SD**	**Min-Max**	***n* (%)**
**Club characteristics**			
Number of sports within the club	2.95 ± 3.53	1-15	
Number of athletes from all sports	327.15 ± 265.16	29-1023	
Number of basketball players	165.15 ± 56.39	29-261	
Number of competitive age-categories	9.30 ± 2.08	4-11	
Number of national titles	8.10 ± 19.40	0-86	
Number of regional titles	7.40 ± 12.11	0-51	
Number of years of the club' basketball section	49.25 ± 34.75	6-110	
**Club infrastructure**			
Own facilities (Yes/No)			8(40.00)/12(60.00)
Complementary equipment			
Gym (Yes/No)			12(60.00)/8(40.00)
Warm-up area (Yes/No)			5(25.00)/15(75.00)
Medical/Physiotherapy office (Yes/No)			14(70.00)/6(30.00)
Hydrotherapy (Yes/No)			2(10.00)/18(90.00)
Video Room (Yes/No)			13(65.00)/7(35.00)
Practices always in club' facilities (Yes/No)			2(10.00)/18(90.00)
**Human resources**			
Number of coaches	11.80 ± 3.97	2-19	
Coaches' certification level			
Number of coaches with level 1	5.95 ± 2.14	2-10	
Number of coaches with level 2	4.10 ± 2.22	0-10	
Number of coaches with level 3	1.75 ± 1.48	0-5	
Staff			
Physician (Yes/No)			4(20.00)/16(80.00)
Psychologist (Yes/No)			1(5.00)/19(95.00)
Physiotherapist (Yes/No)			17(85.00)/3(15.00)
Nutritionist (Yes/No)			1(5.00)/19(95.00)
**Club communication**			
Social media (Yes/No)			20(100.00)/0(0.00)
Radio station or tv/online channel (Yes/No)			4(20.00)/16(80.00)

S2 Table. Parameter estimates (standard-errors) for fixed and random effects for speed shot shooting.

	Model 1	Model 2
**Fixed effects, β**		
Intercept (11 years)	24.88 (0.59)***	21.77 (0.82)***
Age (velocity)	2.64 (0.13)***	4.68 (0.40)***
Age^2 (acceleration)		–0.29 (0.05)***
**Random effects, σ^2**		
Club		
Intercept	1.10 (0.73)^{ns}	1.07 (0.71)^{ns}
Player		
Intercept	26.03 (5.69)***	27.87 (5.88)***
Age	1.27 (0.38)***	1.13 (0.36)**
Covariance (intercept/age, σ_{ia})	–4.54 (1.42)**	–4.55 (1.42)**
Residual		
Intercept	14.43 (0.70)***	14.20 (0.69)***
**Model summary**		
Deviance	7315.97	7287.81
Number of estimated parameters	7	8
Δ in Deviance from previous model (Δ in number of parameters)		28.16 (1)***

ns, non-significant.
** $p < 0.01$
*** $p < 0.001$

S3 Table. Parameter estimates (standard-errors) for fixed and random effects for passing.

	Model 1	Model 2
**Fixed effects, β**		
Intercept (11 years)	65.95 (1.24)***	64.67 (1.61)***
Age (velocity)	8.25 (0.26)***	9.16 (0.79)***
Age^2 (acceleration)		–0.13 (0.11)^{ns}
**Random effects, σ^2**		
Club		
Intercept	7.71 (4.90)^{ns}	7.28 (4.76)^{ns}
Player		
Intercept	66.76 (20.66)**	74.53 (21.60)***
Age	3.03 (1.59)^{ns}	3.19 (1.61)*
Covariance (intercept/age, σ_{ia})	–3.95 (5.45)^{ns}	–5.38 (5.60)^{ns}
Residual		
Intercept	64.93 (3.17)***	64.69 (3.16)***
**Model summary**		
Deviance	9218.22	9216.92
Number of estimated parameters	7	8
Δ in Deviance from previous model (Δ in number of parameters)		1.30 (1)^{ns}

ns, non-significant.
* $p < 0.05$
** $p < 0.01$
*** $p < 0.001$

S4 Table. Parameter estimates (standard-errors) for fixed and random effects for control dribble.

	Model 1	Model 2
**Fixed effects, β**		
Intercept (11 years)	21.38 (0.14)***	22.30 (0.19)***
Age (velocity)	–0.76 (0.03)***	–1.35 (0.09)***
Age^2 (acceleration)		0.08 (0.01)***
**Random effects, σ^2**		
Club		
Intercept	0.03 (0.03)^{ns}	0.03 (0.03)^{ns}
Player		
Intercept	1.80 (0.36)***	1.74 (0.35)***
Age	0.07 (0.02)***	0.05 (0.02)*
Covariance (intercept/age, σ_{ia})	–0.30 (0.08)***	–0.24 (0.08)**
Residual		
Intercept	0.78 (0.04)***	0.77 (0.04)***
**Model summary**		
Deviance	3705.94	3663.07
Number of estimated parameters	7	8
Δ in Deviance from previous model (Δ in number of parameters)		42.87 (1)***

ns, non-significant.
* $p < 0.05$
** $p < 0.01$
*** $p < 0.001$

S5 Table. Parameter estimates (standard-errors) for fixed and random effects for defensive movement.

	Model 1	Model 2
**Fixed effects, β**		
Intercept (11 years)	26.20 (0.20)***	28.47 (0.25)***
Age (velocity)	–1.15 (0.04)***	–2.55 (0.11)***
Age^2 (acceleration)		0.19 (0.01)***
**Random effects, σ^2**		
Club		
Intercept	0.13 (0.08)^{ns}	0.11 (0.07)^{ns}
Player		
Intercept	4.47 (0.71)***	4.12 (0.67)***
Age	0.21 (0.04)***	0.10 (0.03)***
Covariance (intercept/age, σ_{ia})	–0.84 (0.17)***	–0.57 (0.14)***
Residual		
Intercept	1.08 (0.05)***	0.99 (0.05)***
**Model summary**		
Deviance	4253.18	4096.70
Number of estimated parameters	7	8
Δ in Deviance from previous model (Δ in number of parameters)		156.48 (1)***

ns, non-significant.
*** $p < 0.001$

S6 Table. Parameter estimates (standard-errors) for fixed and random effects for slalom sprint.

	Model 1	Model 2
**Fixed effects, β**		
Intercept (11 years)	18.34 (0.10)***	19.21 (0.15)***
Age (velocity)	–0.54 (0.02)***	–1.10 (0.07)***
Age^2 (acceleration)		0.08 (0.01)***
**Random effects, σ^2**		
Club		
Intercept	0.01 (0.01)^{ns}	0.01 (0.02)^{ns}
Player		
Intercept	0.92 (0.19)***	1.15 (0.21)***
Age	0.04 (0.01)**	0.04 (0.01)***
Covariance (intercept/age, σ_{ia})	–0.15 (0.05)**	–0.18 (0.05)***
Residual		
Intercept	0.45 (0.02)***	0.42 (0.02)***
**Model summary**		
Deviance	3030.68	2966.55
Number of estimated parameters	7	8
Δ in Deviance from previous model (Δ in number of parameters)		64.13 (1)***

ns, non-significant.
** $p < 0.01$
*** $p < 0.001$

S7 Table. Parameter estimates (standard-errors) for fixed and random effects for slalom dribble.

	Model 1	Model 2
**Fixed effects, β**		
Intercept (11 years)	19.61 (0.13)***	20.85 (0.17)***
Age (velocity)	–0.64 (0.03)***	–1.43 (0.08)***
Age^2 (acceleration)		0.11 (0.01)***
**Random effects, σ^2**		
Club		
Intercept	0.03 (0.03)^{ns}	0.04 (0.03)^{ns}
Player		
Intercept	1.53 (0.29)***	1.51 (0.28)***
Age	0.08 (0.02)***	0.05 (0.02)***
Covariance (intercept/age, σ_{ia})	–0.29 (0.07)***	–0.23 (0.06)***
Residual		
Intercept	0.58 (0.03)***	0.54 (0.03)***
**Model summary**		
Deviance	3379.93	3281.09
Number of estimated parameters	7	8
Δ in Deviance from previous model (Δ in number of parameters)		98.84 (1)***

ns, non-significant.
*** $p < 0.001$
